# SP^5^: Improving Protein Fold Recognition by Using Torsion Angle Profiles and Profile-Based Gap Penalty Model

**DOI:** 10.1371/journal.pone.0002325

**Published:** 2008-06-04

**Authors:** Wei Zhang, Song Liu, Yaoqi Zhou

**Affiliations:** 1 Indiana University School of Informatics and Center for Computational Biology and Bioinformatics, Indiana University School of Medicine, Indiana University-Purdue University Indianapolis, Indianapolis, Indiana, United States of America; 2 Institute of Applied Physics and Computational Mathematics, Beijing, People's Republic of China; 3 Department of Biostatistics, Center of Excellence in Bioinformatics & Life Sciences, University at Buffalo, State University of New York, Buffalo, New York, United States of America; 4 Department of Biostatistics, Roswell Park Cancer Institute, Buffalo, New York, United States of America; 5 Howard Hughes Medical Institute Center for Single Molecule Biophysics, Department of Physiology & Biophysics, University at Buffalo, State University of New York, Buffalo, New York, United States of America; University College London, United Kingdom

## Abstract

How to recognize the structural fold of a protein is one of the challenges in protein structure prediction. We have developed a series of single (non-consensus) methods (SPARKS, SP^2^, SP^3^, SP^4^) that are based on weighted matching of two to four sequence and structure-based profiles. There is a robust improvement of the accuracy and sensitivity of fold recognition as the number of matching profiles increases. Here, we introduce a new profile-profile comparison term based on real-value dihedral torsion angles. Together with updated real-value solvent accessibility profile and a new variable gap-penalty model based on fractional power of insertion/deletion profiles, the new method (SP^5^) leads to a robust improvement over previous SP method. There is a 2% absolute increase (5% relative improvement) in alignment accuracy over SP^4^ based on two independent benchmarks. Moreover, SP^5^ makes 7% absolute increase (22% relative improvement) in success rate of recognizing correct structural folds, and 32% relative improvement in model accuracy of models within the same fold in Lindahl benchmark. In addition, modeling accuracy of top-1 ranked models is improved by 12% over SP^4^ for the difficult targets in CASP 7 test set. These results highlight the importance of harnessing predicted structural properties in challenging remote-homolog recognition. The SP^5^ server is available at http://sparks.informatics.iupui.edu.

## Introduction

Fold recognition refers to recognizing the structural fold of a protein, given its sequence information. Fold recognition is one of the key bottlenecks for protein structure predictions as the protein data bank now appears to contain the complete (or near complete) set for all possible structural folds of proteins, at least for small domain proteins [1], [2].

Recently completed assessment of automated servers for protein structure prediction (CASP 7) [3] reveals the power of post-treatment of models predicted by individual fold recognition methods through consensus predictions (For example, ROBETTA [4], Pmodeller6 [5], Fams-ace [6]) and/or constrained template-fragment recombination and refinement (For example, Chunk-TASSER [7], I-TASSER [8]). The prediction quality of these methods, however, relies heavily on the accuracy of initial models generated by individual fold recognition methods in the first step. Another observation is that the accuracy of top single servers can rival with most consensus methods. Thus, developing and/or improving individual methods are critically important for further advancement of the accuracy of fold recognition and structure prediction.

We have developed a series of single fold-recognition methods (SPARKS, SP^2^, SP^3^, SP^4^) that are based on weighted matching of multiple profiles that include sequence profiles generated from multiple sequence alignment [9], predicted versus actual secondary structures [10], [11], knowledge-based profile (single-body) score function [10], depth-dependent sequence profiles derived from template structures [11], and predicted versus actual solvent accessible surface area [12]. There is a robust improvement of the accuracy and sensitivity of fold recognition as the number of matching profiles increases [10], [11], and [12]. SPARKS, SP^3^, and SP^4^ were ranked among the top performers for automatic servers in recent CASP 6 [13], [14] and 7 [12], [3]. This exemplifies the importance and effectiveness of multiple-dimensional use of the structural information of templates in developing fold-recognition techniques.

In this paper, we introduce the fifth “dimension” for fold recognition by incorporating predicted backbone torsion angles (SP^5^). The backbone torsion angles (φ and ψ) are two rotation angles about the C_α_ – N bond (φ) and the C_α_ – C bond (ψ). Because the polypeptide backbone of a protein is a linked sequence of rigid planar peptide groups, these two angles essentially determine the backbone conformation of proteins. While a three-state classification of secondary structures is a coarse-grained one-dimensional representation of local backbone conformation, backbone torsion angles encode the backbone tertiary structure, at least in principle.

Traditionally, dihedral torsion angles are predicted as a few discrete states based on local (fragment) structural patterns using either machine-learning techniques or classification schemes [15]–[22]. However, there were only a few limited applications of predicted angle states to fold recognition [18] and sequence alignment [23]. The former uses torsion-angle states as a replacement of simple three-state secondary structures to build an iterated alignment hidden Markov model [18]. The latter [23] predicts angle states by hidden Markov model and employs the predicted angles to build structural context-based substitution matrices. Here, we propose to match predicted and actual torsion angles as a new profile term in a multi-dimensional profile-profile alignment. This represents a novel use of predicted torsion angles as a complementary to rather than a replacement of secondary structures for fold recognition. The angel profile used in this work is built on a recent advancement in real-value prediction of torsion angles [24]. By taking advantage of angle periodicity and using integrated neural networks, we have obtained ten-fold-cross-validated mean absolute errors of 38° for ψ and 25° for φ [24]. This accuracy of real-value prediction was found comparable to or more accurate than those based on multi-state classification of the φ – ψ map.

In SP^4^, the effect of solvation was taken into consideration by matching the predicted and actual solvent accessibility (SA). The SA profiles are based on two states (exposed and buried) classified according to an arbitrary threshold of 25%. The two-state classification increases the accuracy of prediction by reducing number of states in SA. This is at the cost of losing the detailed fluctuation pattern of SA along the sequence. We recently have developed method (called Real-SPINE) for real value SA prediction, which yields a 10-fold cross-validated Pearson's correlation coefficient (PCC) of 0.74 between predicted and actual solvent SA [25]. We thus have updated the original two-state SA profile with the new real-value one in developing SP^5^ scoring function.

In addition to the torsion angle and real-value SA term, we will introduce a new variable gap-penalty model to replace the original constant gap-penalty model. The new model is based on insertion and deletion probability profiles generated from PSIBLAST. Several studies [26]–[28] have indicated the usefulness of these context-dependent profiles for improving alignment accuracy. Here, we propose an implementation by using insertion and deletion probability profiles to a fractional power.

The above-proposed algorithm leads to the new method called SP^5^. SP^5^ is tested in two alignment benchmarks and two structure-modeling benchmarks. Results suggest a significant improvement of SP^5^ over SP^3^ and SP^4^ in fold recognition.

## Results

### Parameter Optimization by the PREFAB Benchmark

Weight factors and gap parameters in SP^3^ and SP^4^ were optimized by using Prosup benchmark [38]. In this study, we use PREFAB 4.0 to optimize SP^5^ parameters [39]. We use PREFAB because its reference alignment is made from the consensus of two separate structural alignment programs (CE [40] and FSSP [41]) rather than one in Prosup. Ninety one pairs of proteins are randomly selected from PREFAB benchmark, with sequences identity less than 30% from each other. We optimized the parameters for SP^5^ (with new profile-based gap model) by maximizing the percent of matches between the reference alignment in PREFAB and the alignment made SP^5^. The optimization is done by sequential grid-search until further iterations do not improve the alignment accuracy [11]. The final parameters used are *w*_0_ = 5.6, *w*_1_ = 0.68, *s*_shift_ = −0.27, *w*_2ndary_ = 0.52, *w*_struc_ = 0.46, *w*_sa_ = 2.3, *w*_Δ_ = 1.33, with the accuracy of one-to-one match 62.3%.

### Testing Alignment Accuracy by ProSup and SALIGN Benchmarks

The alignment accuracy of the methods trained by PREFAB benchmarks is tested by the ProSup and SALIGN benchmarks. Prosup benchmark, prepared by Sippl's group, consists of 127 pairs of proteins with alignment by structural alignment program Prosup [38]. SALIGN benchmark [42] contains 200 selected pairs with an average pair sharing 20% sequence identity and 65% of structurally equivalent C_α_ atoms superposed with an rmsd of 3.5 Å [42]. Reference alignment is obtained from the structural alignment obtained from the TMalign program [43] [i.e., TM overlap]. The sequence identity between PREFAB training set and test sets SALIGN and Prosup are 18% and 20%, respectively.

Table 1 shows the alignment accuracy of different methods given by different benchmarks along with the standard errors estimated by bootstrap simulation on 10,000 re-sampling of the data. There is a consistent improvement from SP^3^, SP^4^ to SP^5^. The absolute changes range from 1.9% to 2.4% (3.4%) from SP^4^ (SP^3^) to SP^5^ while the relative increases are between 3–5% (5–6%) [SP^5^ relative to SP^4^ (SP^3^)]. These changes are significantly greater than the estimated standard errors. The improvement is remarkable considering the fact that ProSup benchmark was used as the training set to optimize the parameters of SP^3^ [11] and SP^4^ [12].

**Table 1:** The alignment accuracies for Prosup and SALIGN benchmark.

	SP^3^	SP^4^	SP^5^
Prosup^a^	65.3±0.22%^c^	66.8±0.20%	68.7±0.20%
SALIGN^b^	56.3±0.14%	57.3±0.13%	59.7±0.15%

a One-to-one match given by the method and Prosup.

b One-to-one match given by the method and TMalign.

c Mean value and the standard error (estimated by bootstrap simulation on 10,000 re-sampling of the data set).

### Testing Fold Recognition with Lindahl Benchmark

The ultimate purpose of improving alignment is to make more accurate fold recognition and structure prediction. Lindahl Benchmark is a large data set of 976 proteins, with 555, 434, and 321 pairs of proteins in the same family, superfamily, and fold, respectively [44]. However, DSSP [30] failed to produce results for 9 proteins. Thus, the actually used proteins in this study are 967 and the number of proteins in family, superfamily and fold is 550, 430, and 317, respectively. Here, the fold recognition sensitivity of each method is tested by aligning each protein with the rest 966 proteins, and checking whether or not the method can recognize the member of same family, superfamily or fold as the first rank or within the top 5 ranks. Thus, the benchmark tests both modeling accuracy and ranking methods of proposed methods.

Table 2 shows the fraction of correctly recognized match of proteins in the same family, superfamily, fold as first rank or within top 5 rank of the templates given by various SP methods and several other methods. Although many published methods have been applied to this benchmark [45]–[47], [10], we only list most recent ones [10], [11], [48], [12]. This is because of the time dependent nature of sequence database for sequence profiles. For facilitating the comparison within SP methods, we used original sequence profiles from Ref. [11].

**Table 2:** The success rate for recognizing proteins within the same family, superfamily, or fold in the Lindahl benchmark.

Methods	Family only (%)		Superfamily only (%)		Fold only (%)	
	Top 1	Top 5	Top 1	Top 5	Top 1	Top 5
PSI-BLAST	62.4^a^	67.6	16.0	25.8	2.2	9.8
SPARKS^b^	81.6	88.1	52.5	69.1	24.3	47.7
HHpred	82.9	87.1	58.8	70.0	25.2	39.4
FOLDpro^c^	85.0	89.9	55.5	70.0	26.5	48.3
SP^3,^ d	81.6±0.07^h^	86.8±0.06	55.3±0.11	67.7±0.11	28.7±0.14	47.4±0.16
SP^4,^ e	80.9±0.07	86.3±0.06	57.8±0.11	68.9±0.11	30.8±0.15	53.6±0.15
SP^5,^ f	82.4±0.07	87.6±0.06	59.8±0.11	70.0±0.11	37.9±0.15	58.7±0.16
SP^5,^ g	81.6	87.0	59.9	70.2	37.4	58.6

a The percentage in each cell is the fraction of correctly recognized match of proteins in the same fold, super family, and family as first rank or within top 5 rank of the template .

b From Ref. [10].

c From Ref. [48].

d From Ref. [11].

e From Ref. [12].

f This work.

g This work (The 43 proteins with >30% sequence similarity to PREFAB training set are removed).

h The standard error was estimated by bootstrap simulation on 10,000 re-sampling of the data set.

Table 2 indicates that the improvement over SP^3^ and SP^4^ in success rate of fold recognition by SP^5^ exists in all three levels (family, superfamily, and fold). The largest improvement over SP^4^ is observed in fold level (7% absolute increase in Top 1 and 5% absolute increase for the best in Top 5; 22% relative increase in Top 1, 9.5% in top 5). This is somewhat expected because the method was trained for remote homolog recognition (structurally similar protein with less than 30% sequence identity, PREFAB benchmark). Again the relative improvement of SP^5^ over SP^3^ and SP^4^ is significantly larger than the standard errors estimated from bootstrap simulations. We further removed 43 proteins that have >30% sequence identity with the training sequences in the PREFAB benchmark. Their effect on the final result is negligible. For comparison, we also include the results of PSIBLAST [9], SPARKS [10], HHsearch / HHpred [27] and FOLDpro [48]. The performance of SPAKRS and Foldpro was from Ref. [10] and Ref. [48], respectively. We further performed PSIBLAST and HHpred locally with their default parameters. Among all methods listed [9]–[12], [27], [48], SP^5^ method has the highest success rate on the fold level (both first and top 5 ranks) and the superfamily for the first rank.

Above success rates of matching sequences within the same SCOP classification are based on somewhat subjective SCOP definition of family, superfamily and fold [49]. A more direct measurement of accuracy is to calculate the accuracy of the first-ranked model built from the fold-recognition alignment. The model is first built by transferring the C_α_ coordinates of the template structures to the aligned residues in the query sequence. The constructed model is then assessed by using the MaxSub score between the model and the known native structure. MaxSub score [37] between the predicted (model) structure and the native structure is a measure of similarity between 0.0 (no similarity) and 1.0 (perfect similarity). The value is calculated by searching the largest subset of well-superimposed residues (≤3.5 Å). Table 3 reports the MaxSub scores for the models built by SP^3^, SP^4^ and SP^5^ methods averaged over the number of proteins. Again SP^5^ improves over SP^4^ and SP^3^ in all levels. The relative improvement of SP^5^ over SP^4^ in MaxSub score is 1.4%, 3.1% and 32.2% in family, superfamily and fold levels, respectively.

**Table 3:** The model quality of top-1 ranked models in Lindahl benchmark per protein.

	Total^a^	Family^b^	Superfamily^c^	Fold^d^
SP^3^	0.358 (±0.03%)^e^	0.529 (±0.05%)	0.232 (±0.05%)	0.107 (±0.05%)
SP^4^	0.361 (±0.03%)	0.532 (±0.05%)	0.251 (±0.05%)	0.116 (±0.05%)
SP^5^	0.374 (±0.03%)	0.538 (±0.05%)	0.257 (±0.05%)	0.153 (±0.06%)

a All 976 proteins.

b Family only.

c Superfamily only.

d Fold only.

e The mean MaxSub score and the standard error (estimated by bootstrap simulation on 10,000 re-sampling of the data set) for the first-ranked models.

### CASP7 test set

We use CASP 7 targets [50] as an additional test set for SP5 method. The test set consists of 95 targets and was released between May and July of 2006. The 95 targets were officially classified into 109 template-based-modeling (TBM) domains and 19 free-modeling (FM) domains, based on whether or not the structurally similar template (deposited in PDB) had been identified and used in prediction.

We test SP^3^, SP^4^ and SP^5^ methods on the CASP7 test set. The template library for SP methods was built in the same way. This was done by using the 40% representative domains of SCOP 1.61. The entire chains of multiple-domain proteins are also contained in the library. The library was then updated with new proteins released after SCOP 1.61 if they have less than 40% sequence identity with the sequences already in the library. To make a strict test, we only include template proteins released before May of 2006 for this test, and we also excluded the templates with sequence identity >20% to the query. The performance of different SP method is evaluated by the Maxsub score of the first ranked C_α_ model, which is transferred from the alignment.

Table 4 compares the model quality predicted by SP^3^, SP^4^, and SP^5^. Overall, there is a consistent 3% (5% to 6%) improvement from SP^5^ to SP^4^ (SP^3^) for the CASP 7 targets regardless the evaluation based on domains or full chains. For the 109 TBM domains, SP^5^ is 3% (6%) better than SP^4^ (SP^3^). For the most difficult free-modeling targets, there is a 12% improvement from SP^4^ to SP^5^. This pattern of improvement is consistent with that from Lindahl benchmark. That is, the most significant improvement from SP^4^ to SP^5^ is on the most challenging targets.

**Table 4:** The model quality of top-1 ranked models for CASP7 test set.

	Full^a^	ALL^b^	TBM^c^	FM^d^
SP^3^	0.364 (±0.20%)^e^	0.375 (±0.17%)	0.408 (±0.19%)	0.152 (±0.37%)
SP^4^	0.373 (±0.20%)	0.387 (±0.17%)	0.420 (±0.19%)	0.153 (±0.32%)
SP^5^	0.383 (±0.21%)	0.397 (±0.17%)	0.431 (±0.18%)	0.171 (±0.38%)

a 95 full chain targets.

b All 124 domains (There are 4 targets belonging to both TBM and FM categories).

c 109 Template-based Modeling domains.

d 19 Free Modeling domains.

e The mean Maxsub score and the standard error (estimated by bootstrap simulation on 10,000 re-sampling of the data set) for top 1 model.

## Discussion

This paper reports several significant changes over previously developed SP method: the torsion-angle term for profile-profile matching, real-value-based SA profile, and variable gap-penalty model based on fractional-powered insertion/deletion profiles. We showed that by integrating these new features with existing sequence-derived profile, secondary structure profile, residue depth-dependent structure-based profile, the new method SP^5^ makes a robust improvement over previously developed SP serial methods. Comparing with SP^3^ and SP^4^, there is a 2–6% absolute improvement in one-to-one match of alignment accuracy depending on benchmarks. Application of SP^5^ to the large Lindahl benchmark reveals 1%, 2% and 7% improvements over SP^4^ in success rates in recognizing proteins within the same family, superfamily and fold, respectively. The improvement in recognition leads to 1%, 3% and 32% improvement in modeling accuracy based on the top-1 ranked, family, superfamily and fold-level models, respectively. Additional test on CASP 7 targets yields 3–6% improvement in 109 template-based modeling targets and 12% improvement in 19 free-modeling targets. Thus, SP^5^ marks a significant improvement over SP^3^ and SP^4^ in fold-recognition, as designed.

This paper represents a full exploitation of predicted torsion-angles for fold recognition. Previous similar studies [18], [23] are limited to view discrete torsion-angle states as an expansion of secondary structures. This paper, however, treats predicted angles as complementary information to predicted three-state secondary structures. The two quantities are complementary because three-state secondary structures represent a coarse-grained description of local structures while torsion angles contain detailed local and nonlocal structural information if they are predicted accurately. Indeed, our limited initial test indicates that removing secondary structures from SP^5^ will reduce its alignment accuracy. Obviously, the success of SP^5^ is made possible because of reasonably accurate real-value prediction of torsion angles [24].

Recent progress in sequence alignment and structure prediction has suggested the importance of variable gap penalties in protein sequence alignment [51]. Different form of context (either structure or sequence context or both)-dependent gap-penalty model has been proposed [52], [53]. Employing fractional-powered gap insertion/deletion profiles is another new feature introduced in SP^5^. While these insertion/deletion profiles were used, previously [26]–[28], our trial-and-error analysis indicates that the fractional-powered gap insertion/deletion profiles with a power of 0.1 seem to be more suitable for improving alignment accuracy. However, more systematic comparative studies are needed to check if any other functional forms are more appropriate.

To analyze the usefulness of the new gap model, we made a version of SP^5^ with the previously used gap model and found that new gap model leads to a small but positive increase in alignment accuracy (0.5% in PREFAB, 1.5% in ProSup and 0.1% in SALIGN). Thus, the main contribution for improved ability in fold recognition by SP^5^ is due to introduction of torsion angles.

SP^3^ and SP^4^ were among the top performers in automatic servers in CASP 6 and 7 [13], [12]. It is noted that in CASP7, SP^3^ scored higher than SP^4^ according to GDT-HA, TMscore, and AL0 for all targets. A close examination [12] indicates that SP^4^ is slightly more accurate than SP^3^ in hard targets (FM category), but slightly worse than SP^3^ in other targets (TBM category). This is perhaps because all parameters were optimized for fold recognition targets. On the other hand, SP^4^ performs consistently better than SP^3^ at both FM and TBM categories if the cumulative Z-score is used [12]. The development of SP5 continues our emphasis on searching a more sensitive method for fold recognition. Significant improvement of SP^5^ over SP^4^ and SP^3^ indicates that SP^5^ is among the most accurate automatic servers for fold recognition.

In the SP serial methods, the alignment generated for fold recognition is used directly in modeling. It is quite possible that a separate alignment method optimized for modeling may further improve the accuracy of predicted model. This will be a subject of future studies.

## Methods

### Alignment Score

The alignment score of SP^5^ for aligning query position *i* with the template position *j* is
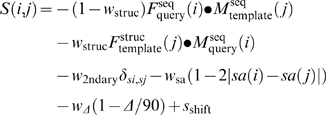
(1)with four weight parameters (*w*_struc_, *w*_2ndary_, *w*_sa_, and *w*_Δ_) and a constant shift *s*_shift_. This score represents weighted matching of five profiles that are described in detail below.

The first term in Eq. (1) is the profile-profile comparison between the sequence profile from the query sequence and that from the template sequence. 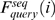 is the sequence-derived frequency profile of the query sequence, 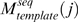 and 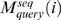 are the sequence-derived log odd profile of the template sequence and that of query sequence, respectively. These sequence profiles are constructed by three iterations of PSIBLAST [9] searching (E value cutoff 0.001) against non-redundant (NR) sequence database, which was filtered to remove low-complexity regions, transmembrane regions, and coiled-coil segments [29].

The second term in Eq. (1) compares the sequence profile from the query sequence and that derived from the template sequence (sequence profiles that would “fit” to the structure). 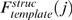 is a depth-dependent sequence profile generated from the sequences of those structural fragments that are similar to 9-residue segment structures of the template [11].

The third term in Eq. (1) measures the difference between the predicted secondary structure of the query sequence and the actual secondary structure of the template. δ*_si_*_,*sj*_ is a simple function of the secondary structure element *si* of the query at sequence position *i* and *sj* of the template at sequence position *j*. δ*_si_*_,*sj*_ = 1 if *si* = *sj* and δ*_si_*_,*sj*_ = −1 if si≠sj. We use a three-state definition of secondary structures (H for helix, E for strand, and C for coil). The secondary structures of templates are from DSSP [30].We have used the convention: (H, G, I) →H, (E, B) →E, and others →C. The secondary structure for query sequences is predicted by SPINE [31]. The first three terms constitute the method SP^3^ [11] except that PSIPRED [29] rather than SPINE [31] was used in SP^3^ to predict the secondary structure of the query sequence. DSSP [30] is used for analyzing template structures because SPINE was trained based on the DSSP definition of secondary structures.

The fourth term in Eq. (1) is the matching score between the predicted solvent accessibility of the query sequence and solvent accessibility of the template structure. *sa(i)* and *sa(j)* are the predicted residue solvent accessibility of query sequence and that of the template structure, respectively. The residue solvent accessibilities of query sequence are predicted by Real-SPINE [25] while residue solvent accessibilities of template structures are calculated from DSSP [30] and normalized by unfolded solvent accessible surface areas [32]. The first four terms constitute the method SP^4^ [33] except that in SP^4^, PSIPRED [29] rather than SPINE [31] was employed to predict the secondary structure of the query sequence, and the real values of solvent accessibility from Real-SPINE [25] rather than two-state classifications by SABLE [34] are used to predict the residue solvent accessibility of the query sequence.

The fifth term in Eq. (1) is a new addition in SP^5^. It characterizes the difference between predicted angles (ψ*(i)* and φ*(i)*) of the query sequence and actual angles (ψ*(j)* and φ*(j)*) of the template structure with
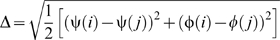
Real values of angles for the query sequence are from Real-SPINE 2.0 [24] while these angles are calculated by DSSP [30] for the template structure. Real-SPINE 2.0 is a method for real-value prediction of torsion angles by using back-propagation neural networks trained with a sliding 21-residue window of sequence profiles, representative amino acid properties, and predicted secondary structures. The ten-fold-cross-validated mean absolute errors are 38° for ψ and 25° for φ, respectively.

### Profile-based Gap Model

SP^3^ [11] and SP^4^ [12] employ a simple secondary-structure dependent gap penalty. No gaps are allowed if *si* = *sj* = α (helix) or *si* = *sj* = β sheet). The gap opening (*w*_0_) and gap extension (*w*_1_) penalties are applied to other regions. In this paper, we construct a profile-based gap model from the multiple sequence alignment made by PSIBLAST [9]. The multiple sequence alignment allows us to calculate the probability of deletion at sequence position *i*, 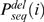, and the probability of insertion at sequence position *i*, 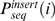, 
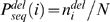
 and 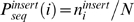 where 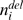, 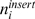, and *N* are number of deletions in sequence position *i*, number of insertions in sequence position *i*, and total number of aligned sequences, respectively.

Thus, we have four profiles: two for query sequences and two for template sequences (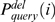, 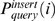, 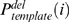, and 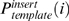)

The gap penalty is calculated as follows. We still use ***w*** **_0_** as the gap opening penalty. The extension gap penalty is modified by 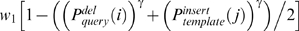 for residue *i* in the query sequence that is aligned with a gap after residue *j* in template. Similarly, the extension gap penalty is modified by 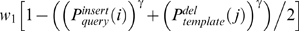 or residue *j* in template that is aligned with a gap after residue *i* in query. Here, ***w*** **_1_** is a to-be-optimized weight factor. Usually, 
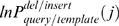
 is an energetic term. Here, we use 
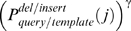
 rather than 
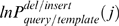
 to avoid singularity at 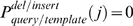. We set γ = 0.1 by trials and errors.

### Dynamic Programming and Template Ranking

Similar to SP^3^ and SP^4^, we used the Smith-Waterman local alignment algorithm [35] to optimize the score that matches the query profiles with template profiles based on Eq. (1) with the revised gaping method described above. Note that the optimization of alignment is to minimize the total alignment score due to the negative signs in Eq. (1).

The templates are ranked based on the difference score between the raw alignment score and the reverse alignment raw score in which the alignment is made with the reversed query sequence [36]. The results of fold-recognition alignment are used to build Cα models based on native template structure. This is done by directly transferring the Cα coordinates of the template structures to the aligned residues in the query sequence. If there is no structural similarity between first two models (defined as zero MaxSub score [37]), templates will be re-ranked by the greater one of two Z-scores, which are calculated based on the raw alignment score normalized by the full alignment length and the non-end-gap alignment length, respectively. Here, the Z-score for a template *i* is given by 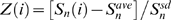, where *ave* and *sd* denotes the average and standard deviation of normalized score for all the templates. This ranking mechanism was based on an empirical observation. We found that ranking based on the difference score between the raw alignment score and the reverse alignment raw score works well only if there is some structural similarity between the top-two ranked models (i.e. a significant structural cluster detected). Otherwise, ranking based on Z-scores works better [11].
